# The extended lipid panel assay: a clinically-deployed high-throughput nuclear magnetic resonance method for the simultaneous measurement of lipids and Apolipoprotein B

**DOI:** 10.1186/s12944-020-01424-2

**Published:** 2020-12-01

**Authors:** Erwin Garcia, Dennis W. Bennett, Margery A. Connelly, Elias J. Jeyarajah, Franklin C. Warf, Irina Shalaurova, Steven P. Matyus, Justyna Wolak-Dinsmore, David N. Oskardmay, Randolph M. Young, Maureen Sampson, Alan T. Remaley, James D. Otvos

**Affiliations:** 1 Laboratory Corporation of America Holdings (LabCorp), Morrisville, NC 27560 USA; 2 LabCorp, Burlington, NC USA; 3 Clinical Center, Dept. Laboratory Medicine, National Institutes of Health, Bethesda, MD USA; 4 Lipoprotein Metabolism Laboratory, Translational Vascular Medicine Branch, National Heart, Lung, and Blood Institute, National Institutes of Health, Bethesda, MD USA

**Keywords:** Lipid panel, Apolipoprotein B, Cardiovascular disease, Nuclear magnetic resonance spectroscopy

## Abstract

**Background:**

Standard lipid panel assays employing chemical/enzymatic methods measure total cholesterol (TC), triglycerides (TG), and high-density lipoprotein cholesterol (HDL-C), from which are calculated estimates of low-density lipoprotein cholesterol (LDL-C). These lipid measures are used universally to guide management of atherosclerotic cardiovascular disease risk. Apolipoprotein B (apoB) is generally acknowledged to be superior to LDL-C for lipid-lowering therapeutic decision-making, but apoB immunoassays are performed relatively infrequently due to the added analytic cost. The aim of this study was to develop and validate the performance of a rapid, high-throughput, reagent-less assay producing an “Extended Lipid Panel” (ELP) that includes apoB, using the Vantera® nuclear magnetic resonance (NMR) analyzer platform already deployed clinically for lipoprotein particle and other testing.

**Methods:**

Partial least squares regression models, using as input a defined region of proton NMR spectra of plasma or serum, were created to simultaneously quantify TC, TG, HDL-C, and apoB. Large training sets (*n* > ~ 1000) of patient sera analyzed independently for lipids and apoB by chemical methods were employed to ensure prediction models reflect the wide lipid compositional diversity of the population. The analytical performance of the NMR ELP assay was comprehensively evaluated.

**Results:**

Excellent agreement was demonstrated between chemically-measured and ELP assay values of TC, TG, HDL-C and apoB with correlation coefficients ranging from 0.980 to 0.997. Within-run precision studies measured using low, medium, and high level serum pools gave coefficients of variation for the 4 analytes ranging from 1.0 to 3.8% for the low, 1.0 to 1.7% for the medium, and 0.9 to 1.3% for the high pools. Corresponding values for within-lab precision over 20 days were 1.4 to 3.6%, 1.2 to 2.3%, and 1.0 to 1.9%, respectively. Independent testing at three sites over 5 days produced highly consistent assay results. No major interference was observed from 38 endogenous or exogenous substances tested.

**Conclusions:**

Extensive assay performance evaluations validate that the NMR ELP assay is efficient, robust, and substantially equivalent to standard chemistry assays for the clinical measurement of lipids and apoB. Routine reporting of apoB alongside standard lipid measures could facilitate more widespread utilization of apoB for clinical decision-making.

**Supplementary Information:**

The online version contains supplementary material available at 10.1186/s12944-020-01424-2.

## Introduction

Lipid panels measure total cholesterol (TC), triglycerides (TG), and high-density lipoprotein cholesterol (HDL-C) and generally also report calculated values of low-density lipoprotein cholesterol (LDL-C) and non-HDL cholesterol (non-HDL-C). They are among the most frequently-ordered laboratory tests owing to clinical practice guidelines that recommend their routine use for assessing and managing risk of atherosclerotic cardiovascular disease (ASCVD) in both primary and secondary prevention settings [1, 2]. By contrast, clinical demand for apolipoprotein B (apoB) testing is much lower despite a consensus that this immunoassay measure of total atherogenic lipoprotein particle number is more directly related to ASCVD risk than LDL-C or non-HDL-C and would provide a better guide to LDL-lowering therapeutic decision-making, particularly in patients with metabolic diseases such as obesity and diabetes [3, 4]. The reason there is not stronger advocacy in guidelines for routine apoB testing is the added analytic cost to patients and the healthcare system. Based on 2019 Centers for Medicare and Medicaid Services (CMS) payment rates, adding apoB to a lipid panel would more than double costs ($21.09 for apoB; $13.39 for lipid panel) [5].

In the clinical laboratory, lipid panel measurements are typically performed using automated enzymatic/colorimetric assays while apoB is measured immunoturbidometrically. While both analytic methods are rapid and sensitive, they require use of multiple reagents and sometimes different analyzers. Also, because they use spectrophotometric detection there is susceptibility to interference from endogenous substances in the sample (e.g., lipemia, hemolysis, icterus). Nuclear magnetic resonance (NMR) spectroscopy offers a potentially attractive alternative means of analysis that utilizes no assay-specific reagents and is minimally susceptible to analytic interferences. The Vantera® NMR analyzer platform is currently deployed clinically for high-throughput *NMR LipoProfile*® testing, providing from a single “scan” (proton NMR spectrum) of a plasma or serum specimen the simultaneous measurement via deconvolution analysis of LDL and HDL particle numbers, lipoprotein subclasses, the Lipoprotein Insulin Resistance Index (LP-IR), the GlycA marker of systemic inflammation, and concentrations of several small molecule metabolites [6–11]. Described here is methodology that uses partial least squares (PLS) regression to extract from the same *NMR LipoProfile* scan the concentrations of TC, TG, HDL-C, and apoB to constitute an “Extended Lipid Panel” (ELP). Extensive validation is provided that accuracy and precision are substantially equivalent to traditional chemical/immunochemical analysis. By eliminating the incremental analytic cost and effort associated with apoB measurement, the NMR ELP assay may help realize the anticipated clinical benefits of more routine use of apoB for therapeutic decision-making.

## Methods

### Buffer and specimens

The buffer was prepared by mixing Na_2_HPO_4_ and CaEDTA (Sigma-Aldrich, St. Louis, MO, USA) at pH 7.4. Serum controls were purchased when commercially available. Pools for studies were generated at LabCorp (Morrisville, NC, USA) from de-identified residual clinical serum samples. When needed, volunteer donors were recruited at LabCorp and each signed an informed consent form. Studies were carried out in accordance with the Declaration of Helsinki and cleared by a local Institutional Review Board. Specimens collected in Greiner tubes (Part # 456293P) were allowed to clot in an upright position for 30 min and centrifuged (3000 rpm) for 10–15 min immediately after clotting. Samples drawn into plain red-top tubes were held upright at room temperature for 45 min to clot and were promptly centrifuged according to manufacturer’s directions. Samples collected in EDTA- and Na-heparin tubes were processed per manufacturer’s specifications.

### NMR and chemical analysis

Proton NMR spectra were collected on 400 M*Hz* Vantera® clinical analyzers at 47 °C with a total acquisition time of 48 s. Detailed spectral acquisition and processing parameters are the same as those described for the *NMR LipoProfile*® test [7, 8, 10]. Chemical lipid and apoB measurements were performed at LabCorp (Burlington, NC, USA) using Roche 8000 c701 analyzers for TC, TG, and HDL-C and Roche/Hitachi cobas c501/502 analyzers for apoB immunoassays.

### Creation of PLS regression models

Separate regression models were built using Wold’s PLS1 method [12] to relate the lipid methyl and methylene region (0.494–1.592 ppm; 1600 data points) of serum *NMR LipoProfile* spectra to the concentrations of TC, TG, HDL-C, and apoB measured by chemical analysis. The same approach has been applied previously, but using small training sets (*n* < 50) not reflective of the wide diversity of the general population [13, 14]. Much larger sample sets (n ≥ ~ 1000) were used here for model development to produce NMR assays with the robustness and accuracy required for clinical use.

In the PLS method, the **X** matrix (1600 data points of the selected NMR spectral region) and the **y** vector (analyte concentration) in the training set were simultaneously modeled to determine the best set of latent variables in **X** to predict **y**. What is meant by “latent variable” is that there is no simple correspondence between analyte concentration and any particular NMR signal(s) or segments of the selected spectral region that would lend itself to linear regression modeling. Instead, the unknown spectral components are generated by the PLS method using non-linear regression to create so-called latent variable proxies for the needed components. These proxies are created from a training set; the larger the training set the better these proxies are modeled. Signals within the PLS spectral region that have no relation to the analyte, such as the sharp signals from lactate and the branched-chain amino acids, do not “interfere” with the analysis because they are not included in the created latent variables.

Each regression model was created using leave-one-out cross-validation, in which a single spectrum is removed from the N spectrum training set and a regression model created from the remaining N-1 spectra [15]. The concentration of the analyte in the sample that produced the absent spectrum is then predicted from the regression model and compared to its chemically-measured value. The process is repeated for every sample in the training set. The predictive ability of the model was then evaluated from the root mean square error of cross validation, RMSE_CV_:

$$ {\mathrm{RMSE}}_{\mathrm{CV}}=\sqrt{\frac{1}{n}}{\sum}_{i=1}^n{\left({e}_i-{p}_i\right)}^2 $$

where *p*_*i*_ is the predicted concentration and *e*_*i*_ is the concentration measured by chemical analysis. The optimum number of latent variables in each model was determined as that giving the minimum value of RMSE_CV_ as the number of latent variables was successively increased. Obvious outliers identified using the DFFITS statistic [16] that might bias model prediction were removed from the training sets prior to generating the final PLS models.

### LDL-C calculation

Estimated LDL-C values reported by the ELP assay software were calculated using the recently-introduced NIH equation [17] as follows:

$$ \mathrm{LDL}\hbox{-} \mathrm{C}\;\left(\mathrm{mg}/\mathrm{dL}\right)=\mathrm{TC}/0.948-\mathrm{HDL}\hbox{-} \mathrm{C}/0.971-\left(\mathrm{TG}/8.56+\mathrm{TG}\;\mathrm{x}\;\mathrm{Non}\hbox{-} \mathrm{HDL}\hbox{-} \mathrm{C}/2140-{\mathrm{TG}}^2/16100\right)-9.44 $$

### Sensitivity, linearity and precision

Sensitivity studies were performed according to Clinical and Laboratory Standards (CLSI) guidelines [18]. Human serum albumin (Sigma-Aldrich, St. Louis, MO, USA) was used as the blank. The albumin solutions (46 mg/mL) were dialyzed overnight (4 °C) against phosphate buffer (pH 7.4) to remove residual citrate. Five serum pools were diluted 20-fold with dialyzed albumin to generate samples containing low levels of lipids to determine the limit of detection (LOD). For the limit of quantification (LOQ), five serum pools were diluted 5-, 10-, or 20-fold using dialyzed albumin solution. All samples were tested in quadruplicate for 3 days on a single Vantera analyzer. LOB, LOD and LOQ were calculated as previously described [7].

Evaluation of linearity was conducted according to CLSI guidelines [19] using serially mixed pools containing low, medium and high levels of each analyte. The low serum pool was prepared by 2-fold dilution of a source pool with delipidated serum proteins. The delipidated proteins were generated by ultracentrifugation (density > 1.21 g/mL). Sera for the high pools were spiked with VLDL (for TG), VLDL+LDL (for TC and apoB) and HDL (for HDL-C) concentrates isolated by ultracentrifugation. Serum pools with lipid values within the low and high pools were selected and used as the medium pool. Mixtures containing lipid values spanning the biological range were prepared by combining volumes of low and medium pools, or medium and high pools. A total of 15 mixtures each for apoB and TC, 17 mixtures for TG and 12 for HDL-C were generated. Four replicates per mixture, as well as the low, medium and high pools were tested on a single analyzer in 1 day.

Within-run and within-laboratory precision were determined in accordance with CLSI guidelines [20]. Three serum pools (low, intermediate and high) were prepared for each analyte by combining selected de-identified residual clinical specimens. Assessment of within-run precision involved testing 20 replicates of each pool in a single day. Within-laboratory precision testing consisted of analyzing the same pools in duplicate twice per day over 20 days (*n* = 80). Mean, standard deviation (SD) and % coefficients of variation (%CV) were calculated for each analyte.

### Reproducibility study

The reproducibility of results from the ELP assay was assessed according to CLSI guidelines [20] at three sites (LabCorp, Morrisville, NC, USA; LabCorp, Burlington, NC, USA; NIH, Bethesda, MD, USA) incorporating serum panels with analyte levels at or around medical decision limits. The panels were tested in duplicate per run, 6 runs per day for 5 days by 1 operator. Three lots of diluent (i.e., phosphate buffer) were incorporated in the runs per day. Each site used one instrument. Mean, SD and %CV were calculated for each site as well as for the combined results of the three testing sites.

### Method comparison study

Comparison of results obtained by chemical analysis and NMR ELP testing was conducted in a manner consistent with CLSI guidelines [21, 22]. To ensure that comparisons for each analyte included sufficient numbers of samples (*n* > 250) that spanned the entire measurement range, de-identified residual clinical specimens were selected for inclusion in the studies on the basis of their previously-measured lipid or apoB values. Different samples were thus used for the study of each analyte. Each sample was split into two tubes and assayed in singlicate by NMR and standard chemistry assays (Roche 8000 c701 for TC, TG and HDL-C; Roche/Hitachi cobas c501/502 for apoB). Non-weighted Deming regression analysis was used to evaluate results comparing the two methods.

### Test for interfering substances

Eight (8) endogenous and 19 common drugs/metabolites were tested for potential analytical interference on TC, TG and HDL-C test results according to CLSI guidelines [23]. The same substances plus an additional 11 drugs were tested for apoB interference. Stock solutions and samples were prepared as previously described [7]. A substance was deemed an interferent if its presence elicited > 10% change in test results. Reasons for such potential interference include direct spectral contributions of the drugs/metabolites to the 1600 data-point analysis region (rare) and possible spectral-altering binding to lipoproteins and other species that contribute to this region.

### Specimen collection tube comparison and stability studies

Blood from 17 donors was drawn into the following four different specimen collection tubes: Greiner tube (serum separator manufactured by Greiner Bio-One, Inc. Part # 456293P, also known as LipoTubes), BD Vacutainer serum tube (red-top, plain serum, no gel barrier), K_2_EDTA plasma tube and Na-heparin plasma tubes. One specimen was diluted (≤50% by volume with phosphate buffer) and 4 were spiked ≤10% *v/v* with VLDL, LDL, HDL stock solutions isolated by ultracentrifugation in order to span the range of normal and abnormal lipid concentrations. Twenty-two samples were tested for each analyte. Results for the plain serum and plasma samples were compared to results for the Greiner tube by linear regression. Bias of > 10% was considered to be a significant difference in results. The study was conducted in accordance with CLSI guidelines [24, 25].

To evaluate ELP analyte stability in different specimen collection tubes, samples were collected from 10 donors to assess stability at room (20–25 °C), refrigerated (2–8 °C) and frozen (− 20 and − 80 °C) temperatures, as well as after different numbers of freeze-thaw cycles. Samples were obtained from four separate studies corresponding to each collection tube type (i.e., Greiner serum, BD Vacutainer serum, K_2_EDTA plasma, Na-heparin plasma). Stability was evaluated over time and the number of freeze-thaw cycles with acceptable differences falling within ±10% of the baseline value (draw day/day 0).

### Testing of reference standards

National Institute of Standards and Technology (NIST) reference material 1951c (frozen human serum) was purchased and analyzed in triplicate using the ELP assay. A set of NIST 1951c material is comprised of two samples corresponding to two levels of TC, TG, HDL-C and LDL-C. The lipid results obtained from the ELP assay were compared to the certified values of TC and TG (measured by isotope dilution gas chromatography-mass spectrometry), and to the reference values of HDL-C (measured by the ultracentrifugation reference method) and LDL-C (measured using the β-quantification reference method).

A blinded set of 3 different serum reference samples from the Centers for Disease Control (CDC) Lipids Standardization Program (LSP) was obtained each quarter and analyzed using the ELP assay. Each reference sample was tested in duplicate 4 times at 3 week intervals. TC, TG, HDL-C and apoB results were sent to the CDC each quarter for evaluation against assigned values determined at a CDC Lipids Reference Laboratory.

## Results

### PLS regression models for quantification of TC, TG, HDL-C and apoB

The lipid methyl and methylene region of proton NMR spectra of human serum encodes detailed information about the concentrations and lipid compositions of the multiplicity of lipoproteins of different size and density that transport lipids in blood [6]. NMR data from this spectral region, obtained from thousands of patient serum samples that had also undergone standard chemical analysis for lipids and apolipoproteins, were used to create separate PLS regression models for TC, TG, HDL-C, and apoB to enable their simultaneous quantification during clinical *NMR LipoProfile* testing.

**Fig. 1:**
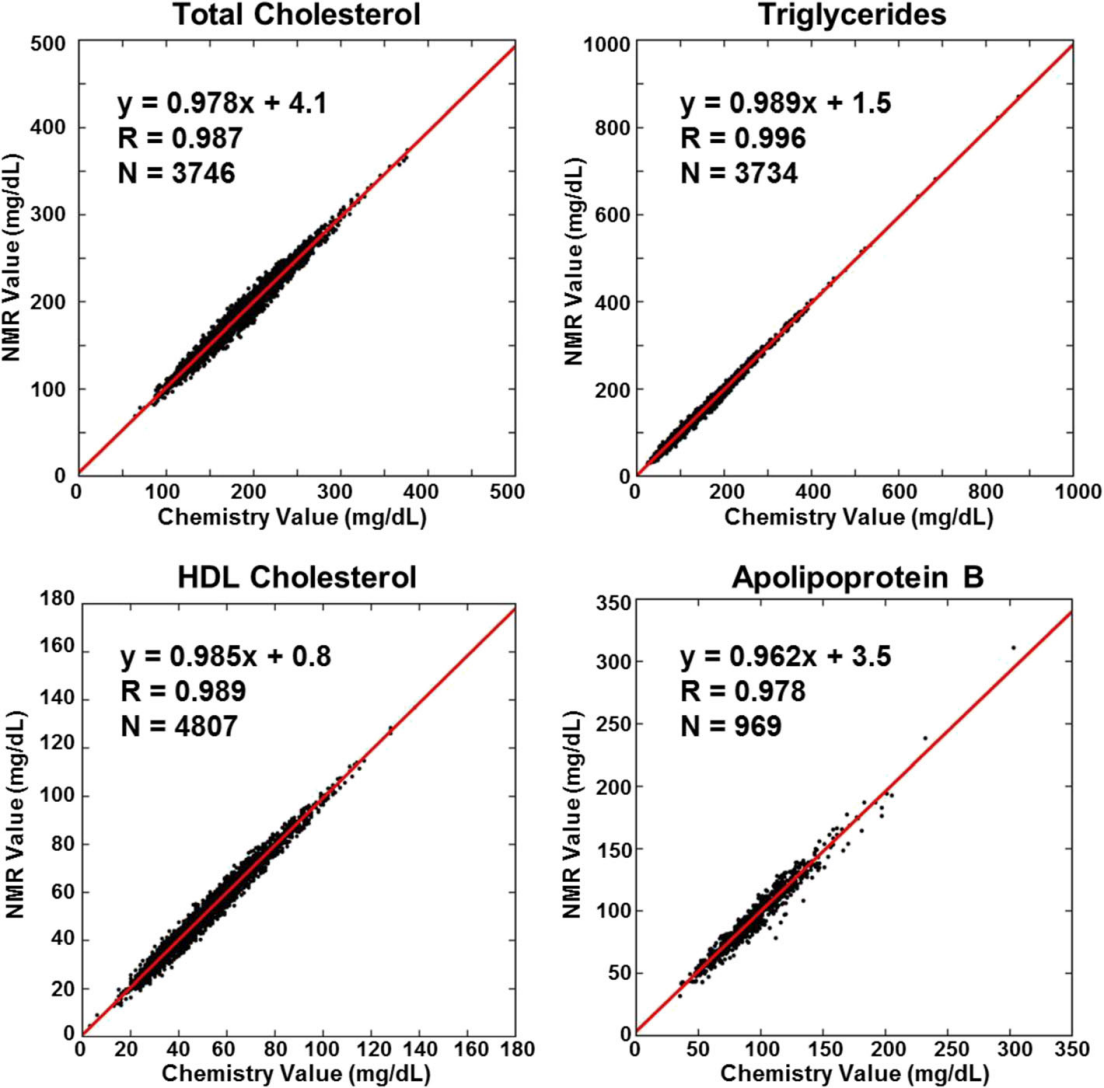
Comparison of chemically-measured and NMR-derived values for the sample sets used to create the PLS regression models

The characteristics of the sample sets used to build the PLS models are given in Table 1. Not only were very large numbers of samples included in each training set (> 3500 for TC, TG, and HDL-C; 969 for apoB), the ranges of lipid and apoB values were also very large so as to encompass the wide diversity of normolipidemic and dyslipidemic samples encountered in clinical practice. Samples with low HDL-C pose an added challenge because the low levels can arise either from low HDL particle numbers and/or from HDL particles with abnormally low amounts of cholesterol per particle as typically found in sera from hypertriglyceridemic individuals. To optimize quantification of HDL-C, two PLS models were created, one for use with normal TG samples (TG < 250 mg/dL) and the other for samples with high TG (≥250 mg/dL). The ELP assay software uses the PLS-determined TG level of the sample to automatically select which of the two PLS models to use for calculating HDL-C. As shown in Table 1 and Fig. 1 for the training sample datasets, the 5 created PLS models produce NMR-derived values of TC, TG, HDL-C, and apoB that strongly correlate with chemically-measured values (*r* ≥ 0.98).

**Table 1:** Sample sets used to build PLS models and the model performance in these sample sets

	Sample Set Characteristics				Model Performance			
PLS Model	N	Mean (mg/dL)	SD (mg/dL)	Range (mg/dL)	Latent Variables	R	RMSE_CV_ (mg/dL)	CV_CV_ %
TC	3746	181	42	64–476	25	0.987	6.67	3.68
TG	3734	127	68	24–886	27	0.996	5.74	4.51
HDL-C (1)	3453	54	16	14–167	31	0.988	2.50	4.60
HDL-C (2)	1354	38	13	3–104	29	0.976	2.28	6.01
ApoB	969	94	27	35–305	23	0.978	5.53	5.91

*TC* Total cholesterol; *TG* Triglycerides; *HDL-C* HDL cholesterol; *ApoB* Apolipoprotein B; HDL-C (1), HDL-C model used for samples with TG < 250 mg/dL; HDL-C (2), HDL-C model used for samples with TG ≥250 mg/dL; R, correlation coefficient; *RMSE*_*CV*_ Root mean square error of cross validation; *CV*_*CV*_ Coefficient of variation of cross validation = RMSE_CV_/Mean.

### Independent validation of ELP assay performance

#### Evaluation of assay sensitivity, linearity and precision

The limits of blank (LOB) were calculated to be 18.9, 11.9, 11.3, and 15.7 mg/dL for TC, TG, HDL-C, and apoB, respectively. The corresponding analytical sensitivity or limits of detection (LOD) were 22.2, 13.8, 12.9, and 17.9 mg/dL, respectively. Testing of several pools with varying analyte concentrations gave functional sensitivity or limits of quantitation (LOQ) of 23.5, 15.2, 12.9, and 17.9 mg/dL, respectively.

**Fig. 2:**
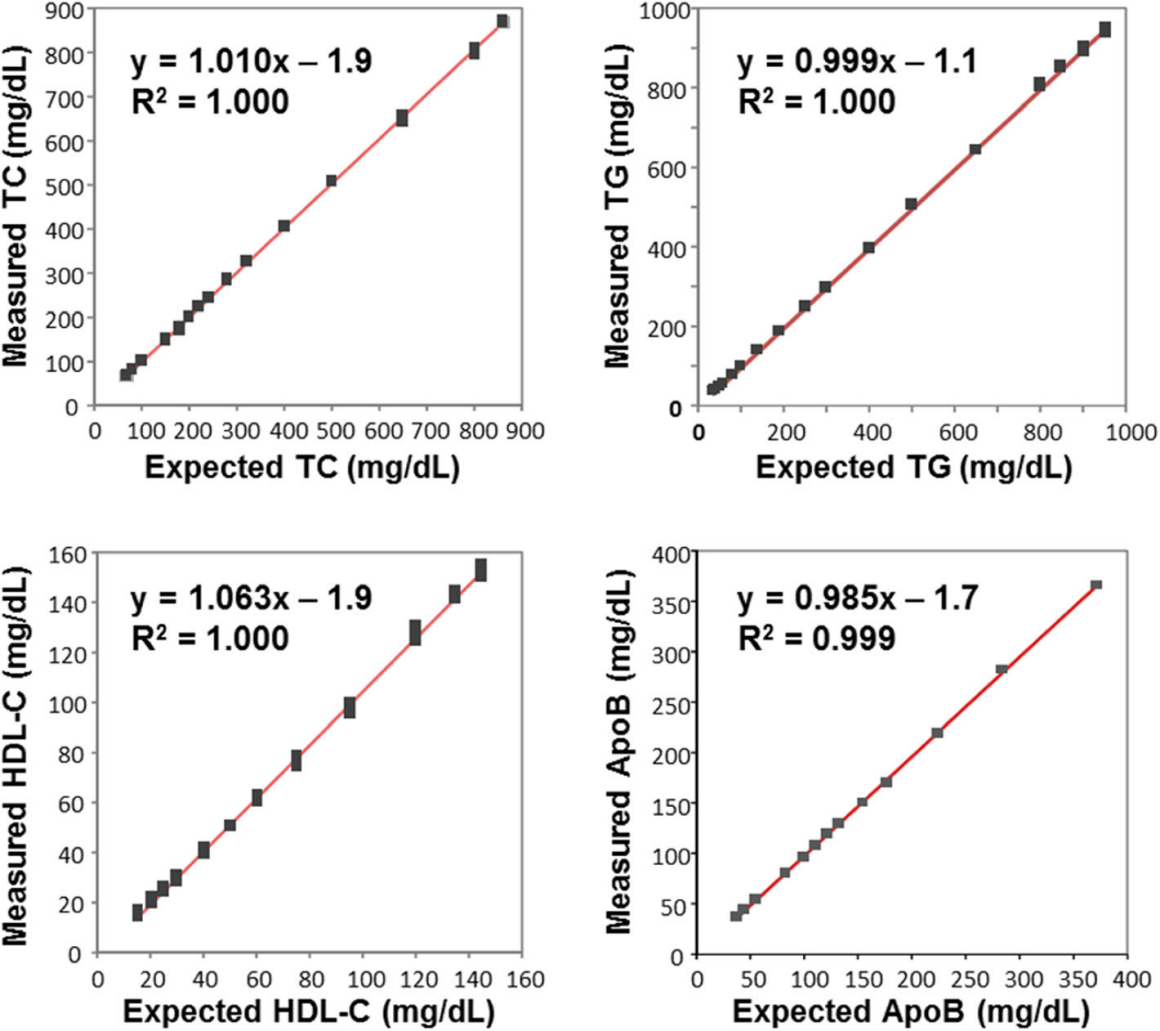
Results of linearity testing of ELP assay analytes

To evaluate linearity over the biological ranges of the ELP analytes, several serum pools with widely varying analyte concentrations were prepared and tested. Plots of expected versus PLS-measured NMR values are shown in Fig. 2, demonstrating excellent linearity over a wide range of concentrations for each ELP analyte.

Serum pools with varying levels (low, medium and high) of each of the analytes were used to evaluate intra-assay (within-run) and inter-assay (within-lab) precision. Results are summarized in Table 2. The CV% for intra-assay precision for all of the analytes ranged from 1.0 to 3.8% for the low, 1.0 to 1.7% for the medium, and 0.9 to 1.3% for the high pools. The CV% for inter-assay precision ranged from 1.4 to 3.6% for the low, 1.2 to 2.3% for the medium, and 1.0 to 1.9% for the high pools.

**Table 2:** Within-run and within-lab precision of ELP assay results

		TC (mg/dL)	TG (mg/dL)	HDL-C (mg/dL)	ApoB (mg/dL)	LDL-C (mg/dL)
**Within-run**^**a**^						
Low	Mean	159.3	128.4	36.6	76.7	57.0
	SD	2.6	1.0	0.9	0.9	2.2
	CV%	1.6	1.0	2.4	1.2	3.8
Medium	Mean	196.3	157.7	49.9	105.5	102.1
	SD	2.6	1.6	0.8	1.1	1.7
	CV%	1.3	1.0	1.6	1.1	1.7
High	Mean	275.8	317.4	91.2	133.8	181.2
	SD	2.6	3.1	1.2	1.4	2.3
	CV%	0.9	1.0	1.3	1.1	1.3
**Within-lab**^**b**^						
Low	Mean	166.6	130.6	36.7	78.9	58.4
	SD	2.7	1.8	1.0	1.9	2.1
	CV%	1.6	1.4	2.8	2.4	3.6
Medium	Mean	197.1	160.9	49.3	109.4	104.4
	SD	2.8	1.9	1.2	2.4	2.1
	CV%	1.4	1.2	2.3	2.2	2.0
High	Mean	279.6	320.3	91.7	137.9	184.9
	SD	3.2	3.1	1.3	2.6	2.6
	CV%	1.1	1.0	1.4	1.9	1.4

^a^ Serum pools tested in 1 run of 20 replicates. ^b^ Serum pools tested in 2 runs of duplicates per day for 20 days (*n* = 80 per analyte). *TC* Total cholesterol; *TG* Triglycerides; *HDL-C* HDL cholesterol; *ApoB* Apolipoprotein B; *CV%* Coefficient of variation expressed as percent

#### Reproducibility of results generated in three clinical laboratories

The reproducibility of ELP assay results obtained in three clinical laboratory sites was evaluated using serum pools with analyte levels at or around their established medical decision limits. Results shown in Supplemental Table 1 (see Additional file 1) indicate very good agreement of ELP analyte concentrations obtained on different Vantera analyzers at the 3 sites, with CV% values generally below 5%.

#### Method comparison

**Fig. 3:**
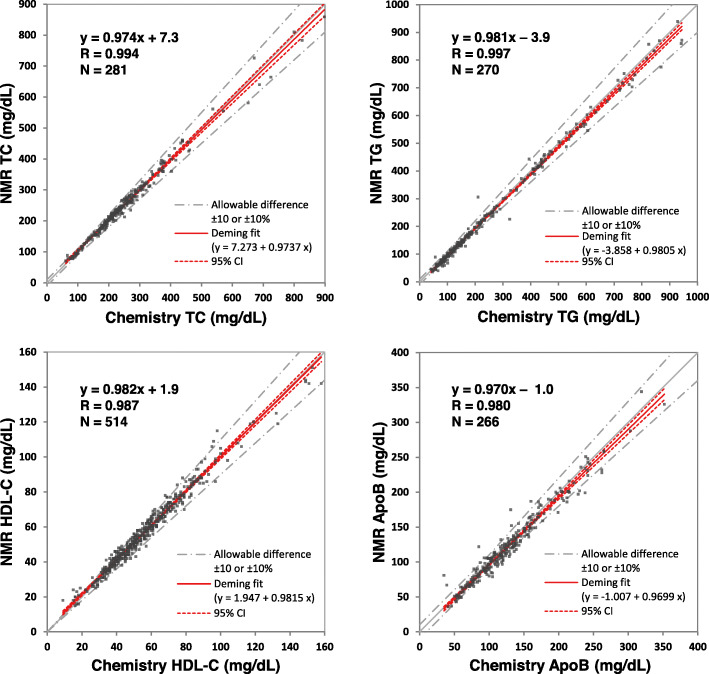
Results of method comparison study evaluating the relations of chemically-measured and NMR-derived values of ELP assay analytes

Independent sets of clinical serum specimens were analyzed to compare results generated by the NMR ELP assay and standard chemistry assays. The method comparison study included 281 samples for TC, 270 for TG, 514 for HDL-C and 266 for apoB, each tested in singlicate. Concentrations obtained by NMR and chemistry testing were highly correlated (r ≥ 0.98) with slopes ranging from 0.970 to 0.982 and intercepts from − 3.9 to 7.3 mg/dL (Fig. 3).

Taking into account the LOQ, linearity and method comparison results, the reportable ranges for the analytes measured by the ELP assay are 66 ─ 868 mg/dL for TC, 35 ─ 950 mg/dL for TG, 14 ─ 152 mg/dL for HDL-C, and 35 ─ 366 mg/dL for apoB. In a clinical testing environment, values outside of these measurement ranges are reported as less than or greater than the lower or upper bounds, respectively, of these ranges.

#### Evaluation of potentially interfering substances

A total of 27 endogenous (e.g. bilirubin, hemoglobin) and exogenous (over-the-counter and prescription drugs) substances were tested in vitro for potential interference with ELP assay HDL-C, TC and TG results, while 38 substances were tested for potential interference with apoB results. All substances were tested at concentrations prescribed by CLSI guidelines. The data in Supplemental Table 2 (see Additional file 1), showing the highest substance concentrations tested that did not elicit interference with TC, TG, HDL-C, apoB and LDL-C results, indicate that none of the substances interfered with the NMR ELP assay at naturally-occurring levels (endogenous) or at therapeutic concentrations (exogenous).

#### Comparison of results from samples obtained using different blood collection tubes

ELP assay results were compared for specimens obtained using the following blood collection tubes: Greiner serum tube (serving as the comparator since it is the preferred collection tube for *NMR LipoProfile* testing), BD Vacutainer serum tubes (red-top), K_2_EDTA plasma tubes, and Na-heparin plasma tubes. Supplemental Table 3 in Additional file 1 shows the results of linear regression analyses comparing ELP values from each collection tube to those obtained using the Greiner tube. Slopes were generally ≥0.95 except for Na-heparin plasma (slope = 0.94), with excellent correlation coefficients (R^2^ = 1.00) for all tube types. No significant bias (> 10%) was observed for the 95% confidence intervals around the correlation slopes or intercepts.

#### Analyte stability in specimens stored at different temperatures

The stability of each analyte reported by the ELP assay at different storage temperatures and freeze-thaw cycles was evaluated in serum and plasma samples. Results were considered acceptable if the means were within 10% of the day 0 mean (Supplementary Table 4 in Additional file 1). All 5 analytes were stable in samples collected in Greiner tubes when stored at room temperature for up to 7 days, refrigerated for up to 14 days, frozen at − 25 to − 10 °C for up to 14 days and frozen at − 70 °C for up to 6 years. All analytes were stable for 5 freeze-thaw cycles, except apoB collected in Greiner tubes which was stable for just one freeze-thaw cycle.

#### Accuracy assessed by analysis of NIST SRM 1951c standard

The accuracy of ELP assay lipid values was assessed by comparing results to the certified/reference values assigned to the NIST 1951c Standard Reference Material (SRM). The purpose of the NIST reference material is to evaluate the accuracy of clinical procedures for determination of TC, TG, HDL-C and LDL-C, as well as validating working or secondary reference materials. As shown in Table 3, ELP assay values were within 4% of the certified/reference values, except for low HDL-C (5.6% bias) and high LDL-C (5.7% bias).

**Table 3:** Comparison of ELP and certified lipid values for the NIST SRM 1951c reference material

	Total Cholesterol (mg/dL)			Triglycerides (mg/dL)			HDL Cholesterol (mg/dL)			LDL Cholesterol (mg/dL)		
Level	ELP value^a^	Ref. value^b^	Bias^c^ %	ELP value	Ref. value	Bias %	ELP value	Ref. value	Bias %	ELP value	Ref. value	Bias %
1	157.3 (2.9)	152.4 (1.8)	3.2	153.0 (1.7)	152.0 (3.2)	0.7	43.3 (1.2)	41.0 (0.9)	5.6	87.0 (1.7)	86.4 (1.4)	0.7
2	243.0 (2.6)	241.4 (2.8)	0.7	139.7 (1.5)	145.4 (3.2)	−3.9	66.3 (1.2)	64.9 (1.7)	2.2	152.0 (1.0)	143.8 (2.1)	5.7

^a^ELP values are means (standard deviation) of triplicate measurement. ^b^Reference values are means (95% confidence intervals). ^c^Bias is percent difference between ELP and reference value

#### Accuracy and precision over time assessed by the CDC lipids standardization program

Participation in the CDC Lipids Standardization Program (LSP) provides external monitoring over time of analytical accuracy and precision of lipid and apolipoprotein testing as performed in clinical laboratory settings. Three blinded LSP serum standards traceable to the CDC Reference Laboratory were obtained quarterly and tested 4 times in duplicate at 3-week intervals. Table 4 summarizes the results of ELP testing for each quarter of 2019. Overall mean bias and CV% over time for TC, TG, HDL-C, and apoB were very low (all < 3%, except 3.6% bias for apoB). Precision and accuracy evaluations for TC, TG, and HDL-C have continuously passed the criteria set by the LSP since ELP assay participation began in 2018 (LSP does not set performance criteria for apoB).

**Table 4:** Accuracy and precision of ELP assay assessed during Q1-Q4 2019 by the CDC Lipids Standardization Program

	Total Cholesterol (mg/dL)				Triglycerides (mg/dL)				HDL Cholesterol (mg/dL)				Apolipoprotein B (mg/dL)			
2019 Quarter/ Serum Pool	ELP Value^a^	CDC Target^b^	Bias^c^ %	CV^d^ %	ELP Value	CDC Target	Bias %	CV %	ELP Value	CDC Target	Bias %	CV %	ELP Value	CDC Target	Bias %	CV %
Q1/162	166.9	169.4	−1.5	1.6	88.3	88.5	−0.2	2.9	58.3	55.3	5.4	1.5	83.9	83.7	0.2	2.0
Q1/163	117.5	116.1	1.2	2.0	74.5	73.0	2.1	1.5	42.1	40.8	3.2	3.2	58.4	53.8	8.6	3.3
Q1/171	176.4	176.2	0.1	3.2	104.3	103.2	1.1	3.2	56.9	55.7	2.2	2.0	84.1	86.3	−2.5	2.5
Q2/171	173.4	176.2	−1.6	1.5	102.9	103.2	−0.3	1.9	56.6	55.7	1.6	2.9	83.4	86.3	−3.4	2.0
Q2/172	157.8	160.7	−1.8	3.0	79.8	78.8	1.3	3.4	51.6	51.8	−0.4	4.0	82.9	80.3	3.2	2.8
Q2/485	131.0	131.4	−0.3	3.6	107.9	106.8	1.0	1.8	37.3	34.8	7.2	2.0	72.4	70.3	3.0	2.8
Q3/171	178.8	176.2	1.5	1.8	105.5	103.2	2.2	2.9	57.9	55.7	3.9	1.4	87.0	86.3	0.8	2.6
Q3/172	162.6	160.7	1.2	1.9	82.4	78.8	4.6	2.2	53.0	51.8	2.3	1.7	84.8	80.8	5.0	2.2
Q3/173	184.0	187.9	−2.1	2.3	254.0	251.9	0.8	2.0	36.5	37.3	−2.1	3.3	106.9	104.8	2.0	1.5
Q4/174	152.8	152.6	0.1	1.8	85.4	88.0	−3.0	1.4	52.6	52.4	0.4	3.0	75.9	70.8	7.2	1.8
Q4/175	187.9	189.4	−0.8	1.4	164.5	165.0	−0.3	0.9	54.4	54.4	0.0	1.4	97.1	93.7	3.6	1.2
Q4/A100	182.4	183.5	−0.6	1.8	103.3	105.1	−1.7	1.3	57.3	55.5	3.2	1.8	90.1	87.0	3.6	3.3
Absolute Mean			1.1	2.2			1.6	2.1			2.7	2.4			3.6	2.3

^a^ELP values are the mean of 8 determinations from ELP testing conducted in duplicate at 3 week intervals. ^b^Target values of the CDC reference sera pools. ^c^Bias is the percent difference between ELP and CDC values. ^d^Precision of ELP measurement as given by the coefficient of variation (CV) of the 8 ELP determinations

## Discussion

NMR spectroscopy remains underutilized for routine clinical analysis despite possessing several attractive analytic attributes. Among these are the lack of need for assay-specific reagents or other consumables (except the diluent buffer) as well as for any specimen pretreatment or processing, insensitivity to interferences that impact spectrophotometric chemical analysis, and most importantly, the multiplex yield of NMR measurement enabling simultaneous quantification of many analytes from a single rapid and automated “scan” of blood plasma or serum [7–11]. Clinical *NMR LipoProfile* testing on the Vantera analyzer platform has not fully leveraged these analytic efficiencies because deployment has primarily been for the purpose of low-volume testing for LDL and HDL particle numbers [7, 8]. The ability to use the same *NMR LipoProfile* scan for routine high-volume lipid panel testing would maximize economies of scale and provide the added bonus of delivering from the same measurement concentrations of apoB plus additional cardiometabolic risk markers including glucose, LP-IR, GlycA, and branched-chain amino acids [9–11].

Reported here are the development of PLS regression models that use a defined spectral region of *NMR LipoProfile* scans to produce high-quality Extended Lipid Panels that include concentrations of TC, TG, HDL-C, and apoB. Performance characteristics of the NMR ELP assay were comprehensively evaluated, including within-lab and between-lab assessments of accuracy and precision, measurement stability of specimens obtained from different blood collection tubes stored at varying temperatures, and testing for potential interfering substances. The results of these quality evaluations validate that the ELP assay is robust and substantially equivalent to traditional chemistry assays for routine use in clinical laboratories. The NMR ELP assay received FDA clearance in 2018. Perhaps the best indication of “real world” testing performance over time are the results of ongoing quarterly proficiency testing of the ELP assay by the CDC Lipids Standardization Program, which since initiation in 2018 have continuously met the precision and accuracy criteria for lipid standardization.

What differentiates the present PLS models intended for clinical use from those generated previously for research applications [13, 14, 26, 27] are the much larger sample sets used for model development. This extra effort was considered necessary to ensure the models were representative of the wide diversity of lipid and apoB levels encountered in the general patient population. The strong correlations (R ≥ 0.98) and minimal bias observed in the method comparison studies between NMR-derived and chemically-measured values of all 4 ELP analytes support this contention. Corresponding assay performance data in the literature for the research NMR methods are scarce, making meaningful comparisons difficult. The largest such split-sample comparison we are aware of (*n* = 4661) reported correlation coefficients of 0.84, 0.77, 0.88, and 0.90 for TC, TG, HDL-C and apoB, respectively, for measurements conducted by chemistry assay and the Nightingale NMR metabolomics platform [28].

Potentially the most consequential clinical benefit of the NMR ELP assay is its ability to deliver, along with traditional lipids and without incremental analytic cost or effort, apoB results that are effectively equivalent to those produced by immunoassay (*R* = 0.980). Comparably good agreement between immunoassay apoB and the sum of the particle concentrations of LDL and triglyceride-rich particles measured by *NMR LipoProfile* testing (*R* = 0.988) was reported previously [29]. Debated for decades, the weight of evidence now clearly supports the superiority of apoB over LDL-C or non-HDL-C as the metric best suited for making individualized lipid-lowering treatment decisions [2–4, 30]. The 2019 European guidelines on lipid management summarized expert consensus as follows: “Given the central causal role of apoB-containing lipoproteins in the initiation and progression of atherosclerosis, direct measurement of the circulating concentration of atherogenic apoB-containing lipoproteins to both estimate risk and guide treatment decisions would be ideal.” [2]. What has kept this ideal from being realized in routine clinical practice is the entrenched historical emphasis on cholesterol as the basis of ASCVD risk management decision-making and the positioning of apoB as “optional” because of its added cost and limited clinical accessibility due to restrictive health insurance payment policies. By addressing the analytic cost differential, clinical implementation of the ELP assay may help bring apoB into more mainstream use to the benefit of clinicians, patients, and the healthcare system.

### Study strengths and limitations

A key strength of this study was the availability of very large datasets of NMR and chemical analysis results from patient serum samples measured over time in a large commercial laboratory. This made possible the training of the PLS regression models with data from thousands of specimens, assuring representativeness of the wide diversity of lipid compositional variability in the general population. Another strength enabled by clinical deployment of the NMR ELP assay in multiple laboratories was the collection of validation data to assess “real world” analytical performance. One limitation of the study is the specificity of the derived PLS regression models to *NMR LipoProfile* spectra acquired on the 400 M*Hz* Vantera analyzer platform. Results are therefore not generalizable to clinical NMR measurements conducted on different NMR instrument platforms using different spectral acquisition protocols.

## Conclusions

Extensive analytic performance evaluations demonstrated that the automated high-throughput NMR ELP assay is efficient, robust, and substantially equivalent to standard chemistry assays for the measurement of lipid panels that are enhanced in clinical value by the inclusion of apoB. Since the only remaining barrier to the benefits that apoB testing would bring to ASCVD risk assessment and management is the added cost, the elimination of the need for a separate immunoassay measurement of apoB by substituting the NMR ELP test could hasten the day that lipid panels routinely augmented by apoB become the new standard of care.

## Supplementary Information

**Additional file 1.**

## Data Availability

The datasets used and/or analyzed during the current study are available from the corresponding author on reasonable request.

## Abbreviations

ASCVD: Atherosclerotic cardiovascular disease
apoB: Apolipoprotein B
CDC LSP: Centers for Disease Control Lipids Standardization Program
CLSI: Clinical and Laboratory Standards Institute
CV: Coefficient of variation
ELP: Extended lipid panel
LOB: Limit of blank
LOD: Limit of detection
LOQ: Limit of quantitation
HDL-C: High-density lipoprotein cholesterol
LDL-C: Low-density lipoprotein cholesterol
NIH: National Institutes of Health
NMR: Nuclear magnetic resonance spectroscopy
PLS: Partial least squares
R: Correlation coefficient
TC: Total cholesterol
TG: Triglycerides
