# Supplementary material for: The extended lipid panel assay: a clinically-deployed high-throughput nuclear magnetic resonance method for the simultaneous measurement of lipids and Apolipoprotein B
Source: Lipids Health Dis. 2020 Dec 1;19:247. doi: 10.1186/s12944-020-01424-2 (PMC7709389; doi:10.1186/s12944-020-01424-2)
Supplement: Supplementary file 1 — Additional file 1. [file 12944_2020_1424_MOESM1_ESM.pdf]

Supplemental Table 1 Between-site reproducibility of ELP assay results

		Total Cholesterol (mg/dL)			Triglycerides (mg/dL)			HDL Cholesterol (mg/dL)			Apolipoprotein B (mg/dL)		
Assay Site		<200	200-240	>240	<150	150 - 200	>200	<40	40 - 60	>60	<90	90 - 130	>160
Site 1	Mean	138.4	213.1	314.8	105.6	170.5	295.7	35.8	50.7	77.9	83.0	112.5	162.5
	SD	2.6	2.9	4.3	1.5	1.4	2.3	1.2	1.3	1.4	1.7	2.3	2.4
	CV%	1.9	1.4	1.4	1.4	0.8	0.8	3.4	2.6	1.9	2.2	2.1	1.5
Site 2	Mean	146.0	216.5	320.0	107.1	167.5	296.5	36.9	54.2	81.5	90.8	120.0	169.8
	SD	3.4	3.2	4.0	4.8	6.6	8.0	1.7	1.2	1.6	3.4	4.0	3.8
	CV%	2.5	1.5	1.3	4.5	4.1	2.7	4.9	2.4	2.0	3.8	3.4	2.3
Site 3	Mean	140.9	211.0	311.9	108.2	170.8	296.2	35.3	51.9	79.4	81.6	112.3	160.6
	SD	4.0	4.5	6.4	1.7	3.0	5.3	1.4	1.2	1.4	2.8	2.7	3.9
	CV%	3.0	2.3	2.2	1.7	1.9	1.9	4.0	2.3	1.9	3.5	2.6	2.7
Combined	Mean	141.8	213.5	315.6	107.0	169.6	296.1	36.0	52.3	79.6	85.1	114.9	164.3
	SD	4.6	4.2	6.0	3.2	4.5	5.7	1.6	1.9	2.1	4.9	4.7	5.3
	CV%	3.3	2.0	1.9	3.0	2.7	2.0	4.5	3.7	2.7	5.8	4.3	3.2

Supplemental Table 2 Interfering substance test results, showing highest concentration (mg/dL) eliciting no interference

Substance	Drug name	TC	TG	HDL-C	ApoB	LDL-C
Bilirubin, unconj.	—	20	20	20	20	21
Bilirubin, conj.	—	34	34	34	34	34
Creatinine	—	5.5	5.5	5.5	5.5	5.5
Hemoglobin	—	219	219	219	219	219
Urea	—	263	263	263	263	263
Uric acid	—	24	24	24	24	24
Protein (albumin)	—	5987	5987	7493	5986	7493
Triglycerides (lipemic)	—	500	—	1135	633	508
Atorvastatin	Lipitor	4.9	4.9	4.9	4.9	4.9
Fenofibrate	Tricor	4.5	4.5	4.6	4.5	4.5
Acetylsalicylic acid	Aspirin	66	66	66	66	66
Acetaminophen	Tylenol	20	20	20	20	20
Naproxen sodium	Aleve	42	56	28	28	28
Ibuprofen sodium salt	Advil	59	29	44	44	59
Hydrochlorothiazide	HCT	0.65	0.65	0.65	0.65	0.65
Metoprolol tartrate	Lopressor	1.5	1.5	1.5	1.5	1.5
Nifedipine	Adalat	0.04	0.04	0.04	0.04	0.04
Enalaprilat dihydrate	Vasotec	0.04	0.04	0.04	0.04	0.04
Hydralazine hydrochloride	Apresoline	19	19	19	19	19
Metformin hydrochloride	Glucophage	65	65	65	65	65
Salicylic acid	—	61	61	61	61	61
Clopidogrel hydrogensulfate	Plavix	18	18	18	9.5	18
Furosemide	Lasix	6.3	6.3	6.3	6.3	6.3
Glipizide	Glucotrol	0.23	0.23	0.23	0.23	0.23
Heparin sodium	Lovenox	303.5 U/dL	303.5 U/dL	303.5 U/dL	303.5 U/dL	303.5 U/dL
Isosorbide dinitrate	Isordil	0.02	0.02	0.02	0.02	0.02
Menhaden oil	Fish oil	241	241	242	241	241
Acetylcysteine	Acetadote	—	—	—	125	—
Ampicillin	Principen	—	—	—	6.1	—
Ascorbic acid	Vitamin C	—	—	—	6.2	—
Calcium dobesilate	Doxium	—	—	—	30	—
Cyclosporine	Neoral	—	—	—	6.1	—
Cefoxitin	Mefoxin	—	—	—	70	—
Levodopa (L—DOPA)	Lodosyn	—	—	—	1.6	—
Methyldopa	Aldomet	—	—	—	1.6	—
Metronidazole	Flagyl	—	—	—	12	—
Doxycycline	Doryx	—	—	—	3.1	—
Rifampin (Rifampicin)	Rifadin	—	—	—	34	—

Supplemental Table 3 Comparison of ELP assay results from specimens collected in different tubes*

Analyte	Tube type	R^2	Slope (SE)	Intercept (SE)	Absolute Bias (mg/dL)	Mean %Bias
Total Cholesterol	Plain Serum	1.00	0.99 (0.00)	5.86 (1.15)	2.9	1.8
	EDTA Plasma	1.00	0.98 (0.00)	0.77 (1.19)	-3.8	-1.8
	Heparin Plasma	1.00	0.98 (0.00)	6.34 (1.15)	1.5	1.3
Triglycerides	Plain Serum	1.00	0.97 (0.00)	3.45 (0.97)	-0.6	0.3
	EDTA Plasma	1.00	0.99 (0.01)	-4.46 (1.28)	-6.7	-4.7
	Heparin Plasma	1.00	0.94 (0.00)	4.30 (1.00)	-4.9	-2.3
HDL Cholesterol	Plain Serum	1.00	0.99 (0.01)	1.12 (0.61)	0.5	1.0
	EDTA Plasma	1.00	0.95 (0.01)	1.55 (0.78)	-1.4	-2.0
	Heparin Plasma	1.00	0.98 (0.01)	1.42 (0.61)	0.1	0.6
ApoB	Plain Serum	1.00	0.97 (0.00)	4.17 (0.55)	1.1	1.8
	EDTA Plasma	1.00	0.96 (0.01)	1.92 (0.87)	-2.1	-1.3
	Heparin Plasma	1.00	0.96 (0.01)	8.78 (0.79)	4.6	6.2
LDL Cholesterol	Plain Serum	1.00	0.99 (0.00)	3.54 (0.69)	2.3	2.5
	EDTA Plasma	1.00	0.99 (0.00)	0.08 (0.79)	-1.4	-1.0
	Heparin Plasma	1.00	0.98 (0.00)	3.98 (0.80)	2.1	2.9

*Compared to Greiner tube.

Supplemental Table 4. Stability of ELP results by storage temperature and freeze-thaw cycles

Conditions	TC	TG	HDL-C	ApoB	LDL-C
Controlled room temperature, 18 to 26°C	7 days	7 days*	7 days	7 days	7 days
Refrigerated, 2 to 8°C	14 days	14 days	14 days	14 days	14 days
Frozen, -25 to -10°C	14 days	14 days	14 days	14 days	14 days
Frozen, -70°C¥	6 years	6 years	6 years	6 years	6 years
Freeze-thaw cycles	5 cycles	5 cycles	5 cycles	1 cycle†	5 cycles§

Data shown are for serum specimens collected in Greiner tubes. Stability was the same in plain serum tubes, EDTA plasma tubes and Na-heparin tubes unless otherwise indicated. *At controlled room temperature, TG was stable for 6 days in Na-heparin plasma tubes and 5 days in plain serum tubes; †ApoB was stable for 5 freeze-thaw cycles in Na-heparin plasma tubes, 4 cycles in plain serum tubes and 3 cycles in EDTA plasma tubes; §LDL-C was stable for 4 cycles in EDTA plasma tubes. ¥-No data available for other collection tube types.
